# Deep learning for enhancement of low-resolution and noisy scanning probe microscopy images

**DOI:** 10.3762/bjnano.16.83

**Published:** 2025-07-16

**Authors:** Samuel Gelman, Irit Rosenhek-Goldian, Nir Kampf, Marek Patočka, Maricarmen Rios, Marcos Penedo, Georg Fantner, Amir Beker, Sidney R Cohen, Ido Azuri

**Affiliations:** 1 Department of Life Sciences Core Facilities, Weizmann Institute of Science, Rehovot, 7610001, Israel; 2 Department of Chemical Research Support, Weizmann Institute of Science, Rehovot, 7610001, Israel; 3 École Polytechnique Fédérale de Lausanne, Laboratory for Bio- and Nano-Instrumentation, CH1015 Lausanne, Switzerland; 4 Bina, Weizmann Institute of Science, Rehovot, 7610001, Israel

**Keywords:** atomic force microscopy, deep learning, fast scanning, low resolution, super resolution

## Abstract

In this study, we employed traditional methods and deep learning models to improve resolution and quality of low-resolution AFM images made under standard ambient scanning. Both traditional methods and deep learning models were benchmarked and quantified regarding fidelity, quality, and a survey taken by AFM experts. The deep learning models outperform the traditional methods and yield better results. Additionally, some common AFM artifacts, such as streaking, are present in the ground truth high-resolution images. These artifacts are partially attenuated by the traditional methods but are completely eliminated by the deep learning models. This work shows deep learning models to be superior for super-resolution tasks and enables significant reduction in AFM measurement time, whereby low-pixel-resolution AFM images are enhanced in both resolution and fidelity through deep learning.

## Introduction

The capability of atomic force microscopy (AFM) to achieve high resolution at the nanometer level in plane (*xy*) and at the angstrom level in height (*z*), on a variety of surfaces, is one of its major advantages. AFM topographical imaging enables high-resolution imaging of simple and complex surfaces that capture the sensitive features, details, and information of the surface structure.

Whereas many manifestations of AFM are in use, including remarkable sub-molecular resolution for specialized systems working under low temperatures and high vacuum [1], the majority use remains that performed on commercial instruments working in ambient (or liquid) environments using one of several operating modes [2–3]. Achieving high-resolution images in such cases is hampered by a few shortcomings. First, the scanning speed of traditional AFM is slow, and several minutes are typically required for each scan. Second, AFM scans can contain inherent artifacts in the captured image due to the operating system settings or the sample and its interaction with the tip. In principle, tip–surface contact should be carefully controlled to avoid damage due to these interactions. Scanning distortions due to non-linearities in the scan are also trickier to correct and harder to avoid in fast scanning. Some of these artifacts can be eliminated or attenuated by image processing techniques [4–12]. Another resolution-limiting factor in AFM is the tip size. In the well-established algorithm of blind reconstruction [13], “certainty maps” are provided, showing clearly where the tip does and does not measure each point. The sharper the tip relative to surface features, the more points it can access. Having said that, blind reconstruction has been used to map the surface to scales below the measured image feature size by “erosion” [14]. It is also important to note that recently machine-learning based methods have been applied to blind reconstruction to reconstruct true surface images from AFM images experimentally broadened by the tip [15]. Although these methods can sharpen images and remove certain tip artifacts, they are not as general in that they cannot upscale the image pixel resolution. Neither these nor the methods presented in this work can provide absolutely true information on parts of the surface the tip does not access.

One approach to obtain high-resolution images and overcome the slow scanning times is to apply image manipulation techniques to upscale low-resolution images to high (pixel) resolution. In a set of studies [16–19], traditional methods and deep learning models were successfully used to reconstruct high-resolution AFM images. In those studies, fidelity metrics were applied to quantify the quality of the reconstructed images. The most common fidelity metrics include peak-signal-to-noise-ratio (PSNR), which is based on pixel-to-pixel differences of the reference and reconstructed images and structural similarity index measure (SSIM), which avoids pixel-to-pixel comparison but instead compares changes in structural information between the images. In the research reported here, in addition to image fidelity metrics, we also incorporate “no-reference image quality metrics” that in some cases may be better aligned with the human perception evaluation and can be even inversely related to fidelity metrics [20]. These metrics are based, for example on contrast, texture richness, and feature frequency. This is of heightened importance for AFM images that may suffer from ubiquitous artifacts and blurring effects in their reference images. Specifically, we calculated here the perceptual index (PI) [20], which combines no-reference image quality measures of Ma et al. [21], which relate to human subjective scoring of super-resolution (SR) images, and the Natural Image Quality Evaluator (NIQE) [22], a blind no-reference image quality metric based on the collection of “quality aware” image statistical features. Note that we use the term super-resolution here, as it is accepted in ML terminology, to refer to upscaling of images by at least a factor of four as was done in the research reported here. This is different from the usage in microscopy where the term refers to resolution beyond the classical physical limitations. Importantly, these no-reference metrics do not use reference images, as the name suggests. It means that they can result in an optimal quality score but do not account for the fidelity of the generated image. For this reason, it is always important to combine metrics from both domains, to first assess the fidelity of the reconstructed image with respect to a reference image and then its quality when attempting to quantify the performance of the upscaling methods and models.

An important distinction between this work and other implementations of deep learning for image enhancement is that in many cases, high-resolution images that are obtained from AFM scans are used to generate low-resolution counterparts by down-sampling to images that then serve as input to traditional methods and deep learning models. This approach is widely used in the community, with the advantage that it saves time and resources. That said, low-resolution images formed in that way are not comprised of physically accessed measurement pixels, simplifying both the experimental and computational components, which may affect the reported metrics. Furthermore, deep leaning models, as a rule of thumb, perform better when they are trained on large data sets. Since we had a relatively small number of images, we opted out of training a custom model and instead chose to use state-of-the-art pre-trained deep learning models trained on a large data set of real-world high-quality images.

In this study, we collected data sets from two different relatively complex surfaces that contain unique but pseudo-repeating structures, which are typical of those seen over a wide range of images spanning common materials and biological surfaces. The data sets contain low-resolution images of 128 × 128 pixels each and their counterpart high-resolution images of 512 × 512 pixels, which serve as the ground truth (GT). Importantly, both low- and high-resolution images are real scans of the surface and neither of them were obtained by image manipulation of the other. Then, we applied a set of traditional methods and SR pre-trained deep learning models on the low-resolution AFM images to obtain their enhanced-resolution image pairs. We performed a comparative study between traditional methods and SR pre-trained deep learning models and quantified their validity and performance by fidelity and quality metrics that were further supported by a survey taken by AFM experts. We found that, overall, the pre-trained SR deep learning models accurately retain the information from the low-resolution images when compared to the GT while also resulting in significantly higher image quality compared to the traditional methods. These findings were supported by appropriate statistical testing.

Importantly, some of the high-resolution GT images suffer from common AFM artifacts that rarely appear in their low-resolution counterparts. Hence, an important outcome of this work, besides resolution enhancement of low-resolution images with the SR deep learning models was the elimination of artifacts in the resulting AFM images. This can serve as a non-destructive method to obtain high-quality SR images of sensitive and soft materials.

## Results and Discussion

**Figure 1:**
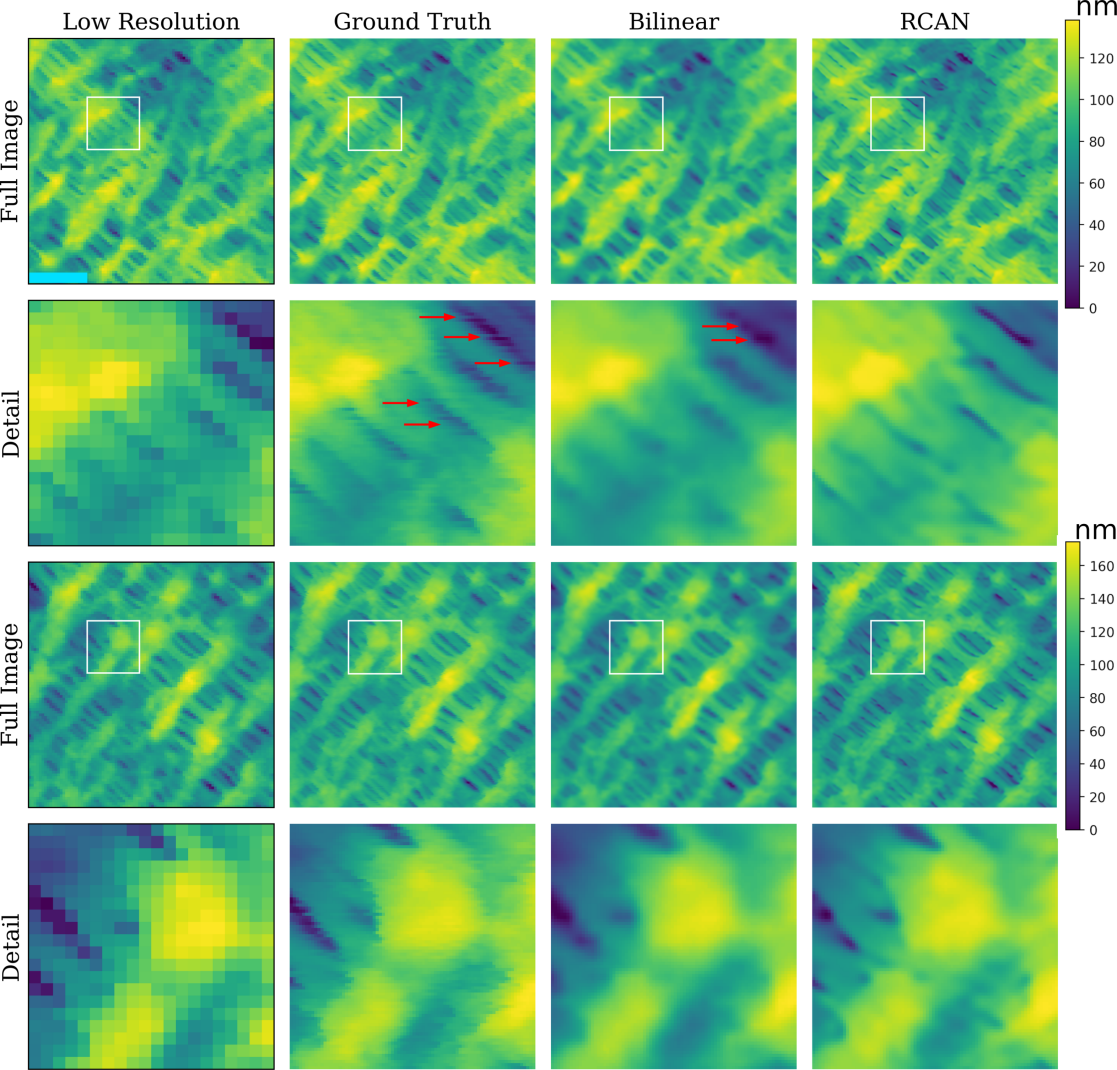
Low- and high-resolution fast-AFM scans of two different locations on a Celgard^®^ 2400 membrane surface, as well as the upscaled images using a traditional method (bilinear interpolation) and a deep learning model (RCAN). The aligned cropped image (94 × 94 and 370 × 370 pixels for low and high resolution, respectively) is displayed along with a corresponding zoom (20 × 20 and 80 × 80 pixels for low and high resolution, respectively) below it (Detail), marked with the white square in the full image. The light blue scale bar in the top-left image corresponds to 0.9 and 0.23 μm on full image and detail, respectively. The red arrows highlight the AFM artifacts, that is, “streaks” in the GT image that may be attenuated in the traditional bilinear method and have been removed in the deep learning model.

**Figure 2:**
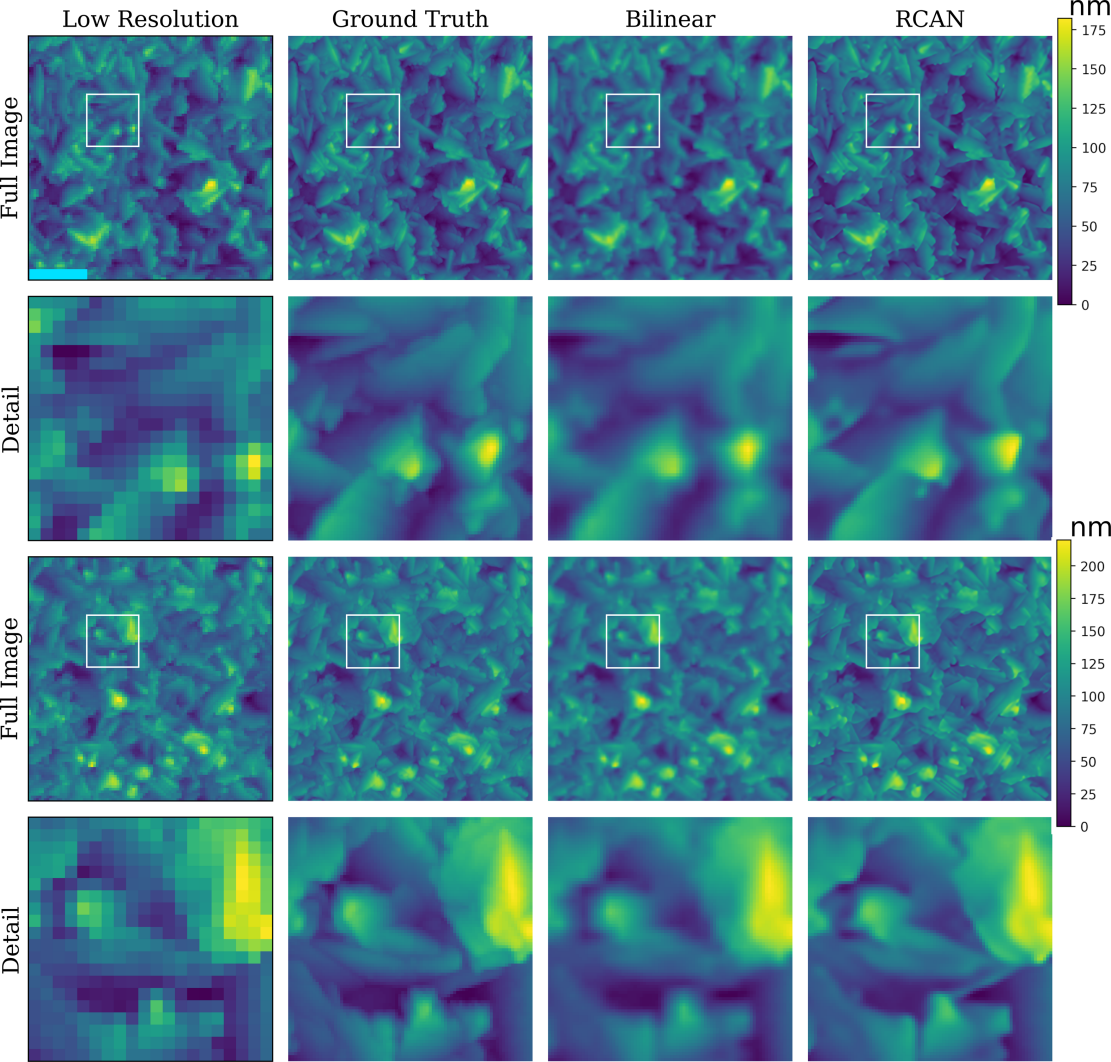
Low- and high-resolution AFM scans of two different locations on a titanium film, as well as the upscaled images using a traditional method (bilinear interpolation) and a deep learning model (RCAN). The aligned cropped image (94 × 94 and 370 × 370 pixels for low and high resolution, respectively) is displayed along with a corresponding zoom (20 × 20 and 80 × 80 pixels for low and high resolution, respectively) below it (Detail), marked with the white square in the full image. The light blue scale bar in the top-left image corresponds to 0.9 and 0.23 μm on full image and detail, respectively.

In this study, 4× upscaled images were obtained from real measured low-resolution AFM images of Celgard^®^ 2400 membrane and high-roughness titanium film used for tip characterization (Figure 1 and Figure 2, respectively). 4× upscaling means upscaling both the *x* and *y* directions and transforming an image of 128 × 128 pixels to 512 × 512 pixels, amounting to 16 times the total number of pixels. We note here that for some modes, such as contact mode or intermittent contact mode, the data is collected continuously and there is no acquisition time price paid for increased pixels in the fast scan direction (here, *x*). However, in some modes, such as peak force and photothermal off-resonance tapping as used here, unless the data is significantly oversampled, decreasing the number of pixels in both *x* and *y* scan directions will lower acquisition time proportionately. We therefore sample here with equal number of pixels in both *x* and *y*. The 4× upscaled images were obtained using traditional methods [23–25] (bilinear, bicubic, and Lanczos4 interpolations) and SR deep learning models [26]. Seven different SR deep learning models were chosen (NinaSR-B0, NinaSR-B1, NinaSR-B2, RCAN, CARN, RDN, and EDSR) [27–31]. The model architectures are built of residual convolutional neural network blocks while each of them integrates a unique algorithmic component (see “Methods”, “Computational Pipeline” section: “Traditional methods and deep learning super-resolution models”). For each low-resolution image, a corresponding AFM high-resolution image was measured and served as the GT. This enabled us to evaluate the 4× upscaled image fidelity with respect to its GT high-resolution AFM image. Due to drift between acquisition at the two different resolutions, it was often necessary to align and crop the images so that corresponding low- (or the model-reconstructed high-resolution image) and high-resolution GT images were perfectly aligned. In addition to reference-based fidelity methods, no-reference methods were used to assess the quality of the 4× upscaled images. In this study, we evaluated the image fidelity and quality obtained with each method and model.

### Image fidelity

**Figure 3:**
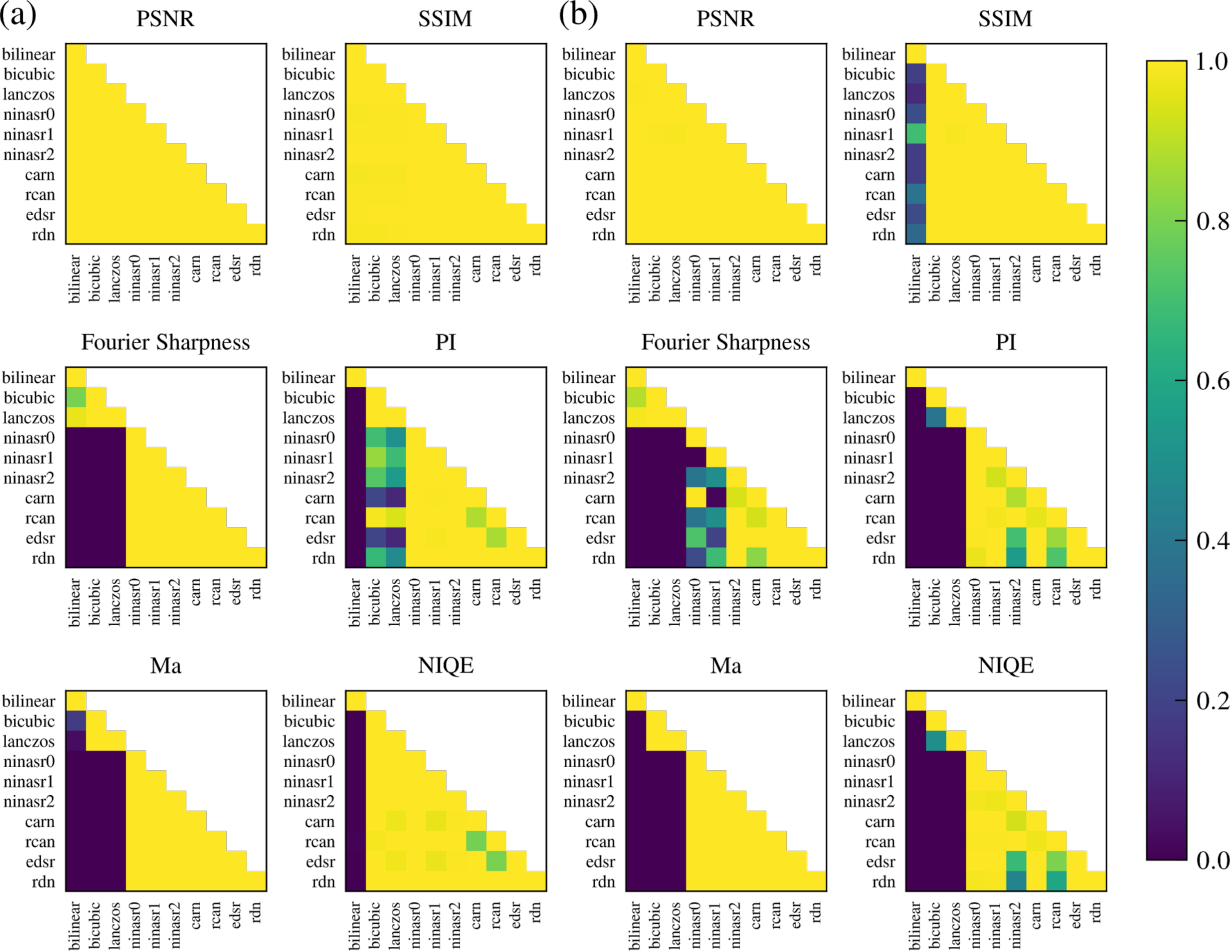
p-Values for six different metrics (PSNR, SSIM, Fourier Sharpness, PI, Ma, and NIQE) for the two data sets, (a) Celgard^®^ 2400 membrane and (b) titanium film, generated using Tukey’s range test. For clarity, only the lower triangular matrix values are presented as the matrices are symmetric. The diagonal represents a value with itself and is therefore always equal to one. Below the diagonal, values closer to zero (blue) represent statistically significant differences between the corresponding method/model performances.

The fidelity of the upscaled images was assessed by using established metrics such as PSNR and SSIM (see further explanation in the Methods section). For both metrics, the 4× upscaled images were compared to the GT. There was no statistically significant difference between the performance of deep learning models compared to the traditional methods, as shown by the p-value Tukey’s range test matrix comparisons in Figure 3 (all p-values are bigger than 0.98, except for that of the bilinear method, which performed worse than the other traditional methods, as well as some of the deep learning models for the SSIM metric with the smallest p-value of 0.12. Importantly, although this value is conventionally considered statistically insignificant, in some cases it could be sufficient, depending on the stringency required). This is also evident from the metrics values in Table 1 and Table 2 for both data sets.

**Table 1:** Comparison of method/model performance across metrics for the Celgard^®^ 2400 membrane data set. Higher values indicate better performance, except for PI and NIQE, where lower values are better. Values in bold represent metrics for which SR deep learning models are statistically significantly better than all traditional methods. Values in parentheses are the range of standard deviation for each metric.

Method/Model	PSNR (4.30–4.76)	SSIM (0.054–0.068)	Fourier sharpness (0.11–0.19)	PI (0.42–0.97)	Ma (0.39–0.66)	NIQE (0.67–1.91)

Bilinear	29.02	0.901	5.194	9.990	2.217	12.20
Bicubic	28.90	0.900	5.246	9.161	2.533	10.85
Lanczos4	28.88	0.900	5.232	9.201	2.603	11.00
NinaSR0	28.58	0.888	**5.483**	8.885	**3.138**	10.91
NinaSR1	28.53	0.889	**5.489**	8.923	**3.173**	11.02
NinaSR2	28.60	0.890	**5.462**	8.896	**3.148**	10.94
CARN	28.57	0.888	**5.465**	8.776	**3.123**	10.67
RCAN	28.56	0.889	**5.485**	8.996	**3.149**	11.14
EDSR	28.57	0.889	**5.465**	8.771	**3.131**	10.67
RDN	28.52	0.887	**5.467**	8.871	**3.167**	10.91

**Table 2:** Comparison of method/model performance across metrics for the titanium film data set. Higher values indicate better performance, except for PI and NIQE, where lower values are better. Values in bold represent metrics for which SR deep learning models are statistically significantly better than all traditional methods. Values in parentheses are the range of standard deviation for each metric.

Method/Model	PSNR (2.22–2.82)	SSIM (0.016–0.022)	Fourier sharpness (0.09–0.16)	PI (0.16–0.25)	Ma (0.24–0.37)	NIQE (0.22–0.57)

Bilinear	32.43	0.915	5.323	9.316	2.892	11.52
Bicubic	33.11	0.93	5.382	7.964	3.6	9.53
Lanczos4	33.17	0.931	5.365	7.829	3.664	9.32
NinaSR0	32.76	0.929	**5.824**	**6.476**	**4.539**	**7.49**
NinaSR1	32.38	0.925	**5.997**	**6.447**	**4.592**	**7.49**
NinaSR2	32.71	0.93	**5.913**	**6.529**	**4.546**	**7.6**
CARN	32.83	0.93	**5.86**	**6.44**	**4.591**	**7.47**
RCAN	32.59	0.928	**5.914**	**6.515**	**4.561**	**7.59**
EDSR	32.65	0.929	**5.895**	**6.42**	**4.58**	**7.42**
RDN	32.61	0.928	**5.924**	**6.408**	**4.573**	**7.39**

Both PSNR and SSIM are based on comparison between the 4× upscaled images and the GT. Since there is no significant statistical difference between the traditional methods and the deep learning models fidelity metrics, image quality metrics remain the key determining factor when comparing any given method or model.

A final comment on fidelity is that there are some cases where small features generated by the deep learning models do not appear in the ground truth. It is not always possible to assess whether these features are genuine or artifacts, as we note that ground truth is also not completely immune to artifacts. These questionable features can be treated as any potential artifact in microscopic images and benefit from verification by independent means, when possible. Minimally, sufficient statistics should be acquired to verify the existence of unusual, important features. A good protocol would be to zoom in and collect a small number of sample images at higher pixel resolution to see whether the features are properly interpreted by the upscaled images. Sometimes, as with all AFM work, it is beneficial to authenticate results using other microscopies such as electron microscopy, or spectroscopic techniques, where relevant.

Importantly, computationally, PSNR average values are very close to 30 for the Celgard^®^ 2400 membrane surface and above 32 for the titanium film, putting them within the acceptable range of values to demonstrate the reliability of the suggested method.

### Image quality

The image quality results show conclusively that the deep learning SR models outperform their traditional counterparts. Figure 1 and Figure 2 provide a comparison of the image quality for both data sets along with the full image as well as image zoom details. These differences in quality between the images obtained with the deep learning models (RACN model) and those obtained with the traditional methods (Bilinear method) are clearly seen.

Quantitative metrics were also used to assess image quality. Natural images are composed of sharply delineated edges, whereas low-quality upscaled images are often characterized by blurred edges. Therefore, measuring the sharpness of an image can act as a proxy for image quality. One way to measure the sharpness is by measuring the gradient between neighboring pixel values.

Additionally, some methods exist that rely on extracting image features. These methods have become increasingly popular, and many variations of such methods have been published [20–22,32–34]. Several such methods were tested; the results were consistent across all of them and are given for PI, Ma, and NIQE. PI is a no-reference image quality metric [20] that incorporates the image feature extraction calculation of Ma and colleagues [21] as well as the NIQE metric [22]. The former relies on human subject studies of SR images and the latter is a blind, no-reference image quality metric based on the collection of “quality aware” image statistical features. PI is thus given by the formula PI = 0.5 × ((10 − Ma) + NIQE).

While Table 1 and Table 2 show the exact values of the different metrics for each method/model (values in bold represent metrics in which all SR deep learning models are statistically significantly better than all traditional methods), Figure 3 illustrates the statistically meaningful differences in performance between the methods/models with respect to image quality metrics, in favor of the deep learning models. While the metrics of the reference-based methods, PSNR and SSIM, show no statistically significant differences between the traditional methods and the deep learning models for image fidelity, the no-reference methods for image quality, Fourier Sharpness, PI, Ma, and NIQE exhibit such differences. Importantly, while for the titanium film data set, all SR deep learning models are statistically significantly better than the traditional methods for all of the no-reference image quality metrics, this is not the case for the Celgard^®^ 2400 data set, for which only the Fourier Sharpness and Ma metrics are statistically significantly better for all SR deep learning models in comparison to the traditional methods. This could arise from the difference between fast and traditional AFM scanning as Celgard^®^ 2400 was measured with fast AFM and titanium film with standard, slow AFM. The fast-scanning is often achieved at the expense of somewhat worse resolution or associated scanning artifacts. The better quality of the latter (titanium film) is reflected in better performance metrics. Nevertheless, this highlights the utility of SR deep learning models for obtaining high-resolution and high-quality upscaled images from low-resolution AFM images. We should also note that the absolute values for PI, Ma, and NIQE are not optimal, since the metrics were designed and constructed for image populations different from AFM images. Nevertheless, the values are adequate for this comparative study.

### Expert survey

**Figure 4:**
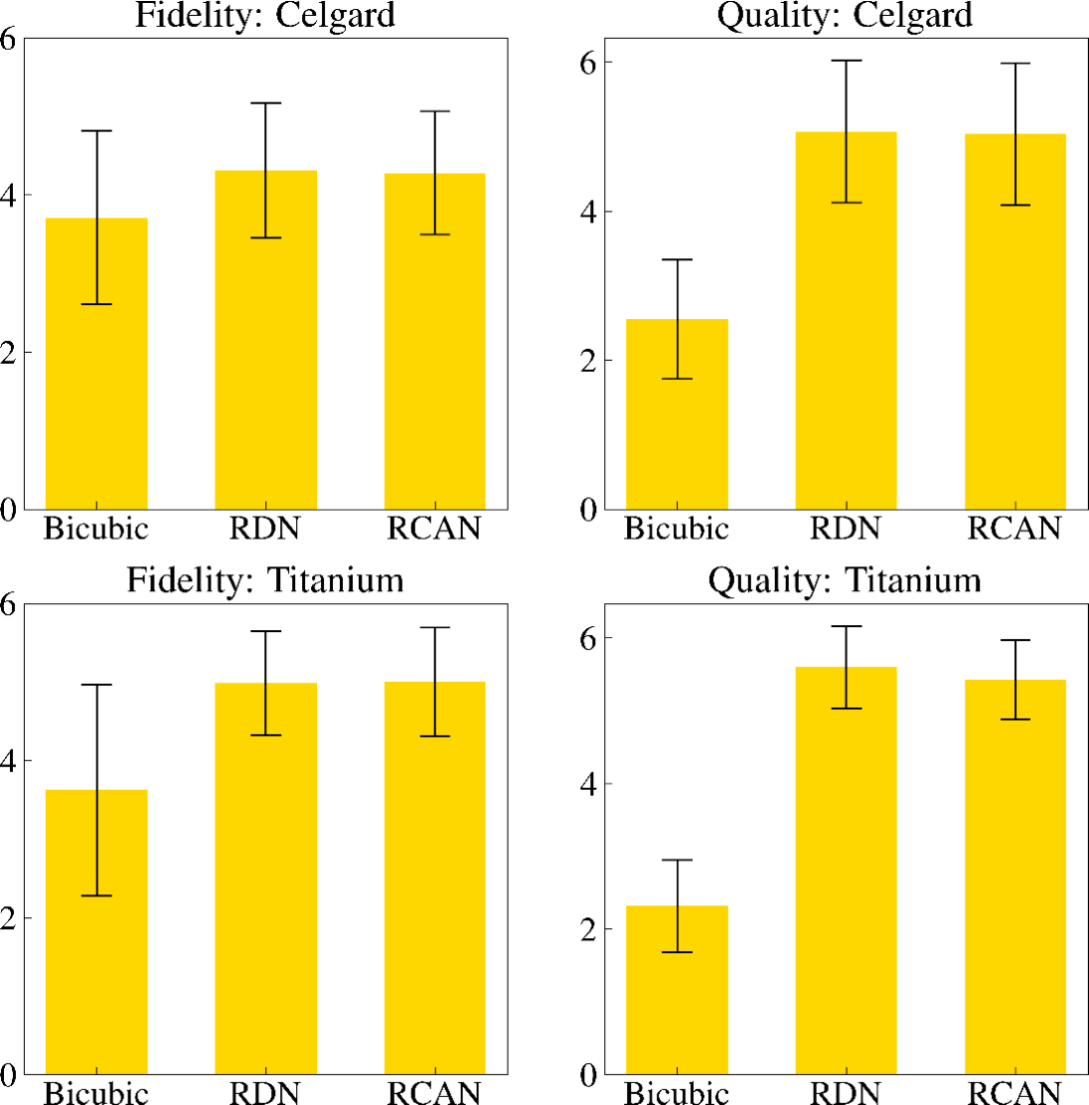
AFM expert survey results. Three experts were asked to judge a blind set of samples and to score each sample’s fidelity and quality compared to the high-resolution ground truth image. The scores are discrete and in the range of 1 (low image fidelity and quality) and 6 (high image fidelity and quality). An example of a data set provided to the surveyors for each sample is presented below in Figure 5. Here, super-resolution models outperformed traditional methods in both quality and fidelity ratings by statistically significant amounts.

AFM experts were presented with a blind test to assess both the fidelity and quality of samples generated using traditional methods and deep learning models (RCAN and RDN models) from both data sets. Figure 4 shows the mean results of the AFM experts and their standard deviations. The experts were asked to rank image fidelity and quality with discrete scores ranging between 1 (for low image fidelity and quality) and 6 (for high image fidelity and quality). The results agree with the other quantitative metrics in that the quality of the deep learning models ranked significantly higher than that of the traditional methods. Importantly, while there was no significant statistical difference in fidelity metrics calculated for the images obtained from traditional methods and deep learning models, in the field experts’ evaluation, there is a statistically significant difference in favor of the deep learning models (with p-values below 2.9 × 10^−7^).

### Pre-trained vs training and fine-tuning deep learning models

For best performance on new, unseen data, deep learning models usually require large amounts of data for training. To avoid this requirement, pre-trained models can be applied. A pre-trained model is a model that has been already trained on the data to perform a given task, here returning SR images from low-resolution images. Then, the pre-trained model can be used for inference without any further fitting procedure on new data such as that used in this research. This approach may work ideally when the target population is similar to the trained data population. Since it was not feasible here to obtain a large amount of data for training a deep learning model, we used pre-trained models supplied by the GitHub repository by Gabriel Gouvine [26]. Importantly, although the models were trained on natural images, which are different from the AFM population images, it yielded satisfactory results in terms of metrics values and AFM field experts. In addition, while EDSR is known to yield the most accurate results, it can be seen that for the pre-trained models, smaller models can yield better results than the EDSR for some of the metrics, as seen in Table 1 and Table 2. Finally, having a custom data set of a specific modality, such as AFM images, may result in an optimized trained or fine-tuned deep learning model which could outperform those employed here. Since our data sets were too small for this purpose, we did not examine this approach here but plan to address it in future work.

### From low- to high-resolution artifact-free AFM images

Some of the high-resolution GT AFM images suffer from common artifacts as can be seen in Figure 1 (red arrows), in particular, in images that were captured at high scanning rates. These artifacts are almost invisible in the low-resolution images. It can be seen that these artifacts do not exist in the 4× upscaled deep learning models that excel in creating sharper natural images. This is in line with the reported ability of the RCAN SR deep learning model to recover images from artifacts [28]. When compared to the traditional methods, these artifacts may disappear as well, but, in some cases, they are only attenuated. This suggests another aspect in which the deep learning models are beneficial. Also, low-resolution images should contain enough information to capture the meaningful image features to ensure that the upscaled high-resolution images will be valid.

## Conclusion

In this study, 4× upscaled high-resolution and high-quality images were obtained from low-resolution AFM images through the use of traditional methods and deep learning models. The effectiveness of these methods and models was then quantified using metrics to gauge the fidelity and quality of their outputs as well as through a survey taken by AFM experts. We found that the deep learning models yield better results in comparison to the traditional methods. In addition, common AFM artifacts such as streaking often appear in the GT high-resolution images. These artifacts are attenuated in the traditional methods while being fully eliminated in the deep learning models. These factors support our conclusion that deep learning models are the method of choice for upsampling low-resolution AFM images to yield high-resolution and high-quality images. Additionally, the application of the suggested procedure can greatly reduce AFM measurement time, enabling the introduction of a faster and more effective procedure into the AFM-based pipeline.

## Methods

The Methods section is composed of Experimental and Computational Pipeline sections.

## Experimental

### AFM image acquisition

For this study we conducted our research on two separate image data sets. For each set, two different image resolutions were captured on overlapping surface areas both at low resolution of 128 × 128 pixels and high resolution of 512 × 512 pixels. The first set is composed of 52 pairs of low- and high-resolution images of a Celgard^®^ 2400 membrane (Celgard, LLC - North Carolina, USA). Images of 5 μm × 5 µm were captured at 8–10 Hz scanning speed, using a fast-scan AFM system, operated by using photothermal off-resonance (at 10 kHz) tapping [35] and small cantilevers, which fit on the base of a commercial MultiMode AFM system (Nanoscope V electronics, Bruker AXS SAS, Santa Barbara, CA). The fast-scanning AFM head and control electronics were designed in-house and built according to the details in [36–38]. Images were acquired using LabView-based software as described in [39]. Scans were made with a silicon tip on a silicon nitride cantilever (FASTSCANC, Bruker). The second set is composed of 25 pairs of low- and high-resolution images of a titanium film, which is used for AFM tip characterization (TipCheck, Aurora Nanodevices, BC, Canada). Images of 5 μm × 5 µm were captured at 1 Hz scanning speed by using a MultiMode AFM with Nanoscope V electronics (Bruker AXS SAS, Santa Barbara, CA) controlled with Nanoscope 9.2 software (Build R2Sr1.130547). Scans were made in PeakForce tapping mode at 2 kHz tapping frequency using a PNP-TRS pyrex-nitride probe formed from silicon nitride (NanoWorld). Images were subject to plane leveling and alignment using Gwyddion 2.62, an open-source software for SPM data analysis [40]. The Gwyddion files were converted to Python .npy files for input to the computational pipeline.

## Computational Pipeline

The research aims to apply traditional methods and SR deep learning models on low-resolution images and increase their resolution to a 4× scale. Increasing the resolution to the 4× scale will make the images the same size as the high-resolution GT images gathered by AFM. A computational pipeline was established to take the low-resolution images, prepare them as input for the various traditional methods and deep learning SR models (Image normalization), apply those methods and models (Traditional methods and deep learning super-resolution models), transform the values to a standard form (Image value transformation), perform alignment along all images (Image alignment), and finally assess the quality of the algorithms using reference and no-reference metrics as well as an expert survey (Metrics) followed by statistical analysis (Statistical analysis).

### Image normalization

The AFM images had a single height channel in units of nanometers. The values of the low-resolution images were normalized to be in the range between zero and one using min–max normalization and expanded equally across RGB color channels as part of the image preprocessing phase for the SR deep learning models. To prepare the normalized low-resolution images for the traditional methods they were converted to pixel values ranging from 0 to 255.

### Traditional methods and deep learning super-resolution models

The choice of SR techniques was aimed at assessing the quality of advanced deep learning approaches and comparing their performance to the traditional methods. Bilinear, bicubic, and Lanczos interpolation were employed using the implementations of Python’s openCV library [41].

The SR deep learning models were all part of the torchSR GitHub repository supplied by Gabriel Gouvine. Five distinct model architectures were used, all of which were built using principles of residual neural network (ResNet) architectures as their backbones. Enhanced deep super-resolution networks (EDSR) make some modifications such as removing batch normalization [31]. Residual dense networks (RDN) employ the use of custom residual dense blocks. This sets them apart from prior dense block techniques, which fail to use additional local dense connections across blocks [30]. Another modification is the cascading residual network (CARN), which uses a cascading mechanism at local and global levels to combine features from both levels [29]. Additionally, SR deep learning models leverage attention along feature channels. This attention is incorporated into residual channel attention blocks, which are stacked to make up deep residual channel attention networks (RCAN) [28]. The torchSR repository also includes a scalable neural network for the SR task, NinaSR [27]. Three different models were supplied (NinaSR-B0, NinaSR-B1, NinaSR-B2), ranging from lighter to heavier sizes. The NinaSR model utilizes local attention blocks and a wide expansion ratio of the residual blocks, and it was initialized using methods adapted from NFNet [27,42].

### Image value transformation

Images generated from the deep learning models were RGB images, and the distribution of pixel values reflected those of the input images. The pixel values of the SR images were clamped to be between zero and one and then were converted to values between 0 and 255. The RGB images were then transformed into greyscale images. The high-resolution GT images were also normalized and converted to values ranging from 0 to 255.

### Image alignment

During the AFM image capture, the low-resolution and high-resolution GT image pairs were taken sequentially over the same area of the sample, resulting in a high image overlap across image pairs. Nevertheless, the overlap location is not perfect due to typical AFM drift. This is also projected on the corresponding images obtained by the traditional methods and SR deep learning models. Hence, it was necessary to first align the obtained images with respect to their corresponding high-resolution GT images to assess the accuracy and quality of the different methods and models used.

We employed a multistep approach for image alignment using OpenCV. First, the scale-invariant feature transform (SIFT) was utilized to detect and describe local features, ensuring invariance to scale and rotation [43]. These features were then matched across images using a brute force matching algorithm, followed by *k*-nearest neighbors to refine the matching process based on Euclidean distance [44]. The best corresponding pixel matches were used to compute the homography matrix, allowing for perspective transformation [45]. The borders of the transformed images and the high-resolution GT images were cropped to ensure precise pixel alignment across the image pairs.

### Metrics

Assessing the effectiveness of traditional methods and SR deep learning models can be divided into two main domains, namely, reference and no-reference metrics. Reference metrics measure the correctness of a method or model by comparing the images obtained from a given method or model against their corresponding GT images. In this study, the images obtained by the different methods and models were compared to their experimentally captured GT counterparts.

No-reference metrics measure the quality of the images obtained by different methods and models. Such metrics try to reflect quality as interpreted by human perception. They often extract features from an image and transform them into a calculated metric. For example, calculating the gradients of an image enables the calculation of edge magnitudes in the image and consequently its sharpness. As the domain name suggests, these metrics do not rely on the presence of GT reference images. These metrics can score optimally but do not account for the fidelity of the generated image. For this reason, it is always important to use methods from both domains to first assess the fidelity of an image using reference metrics and then its quality when attempting to quantify the performance upscaling methods and models.

In addition to the use of algorithmic assessments, a blind and subjective evaluation was conducted, polling AFM experts for their opinions on both image fidelity and image quality.

#### Reference metrics

The first comparison of the SR images to the GT references was to take the peak-signal-to-noise ratio (PSNR). This is done by taking the logarithm of the maximum value of the reference and dividing that by the root-mean squared error between the image and the reference. The PSNR is then multiplied by a coefficient to conveniently scale the metrics output to the decibel scale.

Other methods attempted to avoid pixel-to-pixel differences and instead assess image similarity on a structural basis. This was the motivation for formulating the structural similarity index measure (SSIM) [46]. SSIM has become a widely used reference metric when quantifying image fidelity.

#### No-reference metrics

One intuitive approach to quantify the quality of an image is calculating the sharpness of that image. In some sense, sharpness quantifies the clearness of an image. Nonetheless, high sharpness values can be due to the spikes typical of noise, and values should be interpreted carefully. One way to determine sharpness is by calculating first-order derivatives (gradients) of pixel values along the horizontal and vertical axes of the image (*x*, *y*) and averaging the absolute value of the gradients. A second approach is to transform the image to the frequency domain with the discrete Fourier transform (DFT) when high-frequency regions correspond to the sharp edges. Also here, high-frequency regions may represent noise, and values should be interpreted carefully. Here, we calculated the Fourier sharpness by the mean of the logarithm of the absolute value shifted DFT.

Additionally, there are other no-reference metrics that rely on image features extracted from the images [20–22,33–34]. In this study, the PI, Ma, and NIQE methods were used as no-reference metrics [20–22]. In Ma, low-level statistical features are extracted from super-resolution images in the spatial and frequency domains. The super-resolution images are scored by humans to reflect the human visual perception. Then, a regression model is trained to map between the extracted low-level statistical features and the human scores and is available for inference on new images. NIQE, in contrast, is not based on human scoring and, in that sense, is a completely blind, no-reference, image quality metric. It extracts statistical features in the spatial domain that are associated with image quality from a collection of natural images. A multivariate Gaussian model is fitted to the extracted features and serves as a reference model. Then, the same procedure is applied to new images, resulting in a second fit. The fit is compared to the reference fit, and the deviations from it yield the NIQE score. PI is derived from the combination of Ma and NIQE and is given explicitly by PI = 0.5 × ((10 − Ma) + NIQE).

### Experts survey

**Figure 5:**
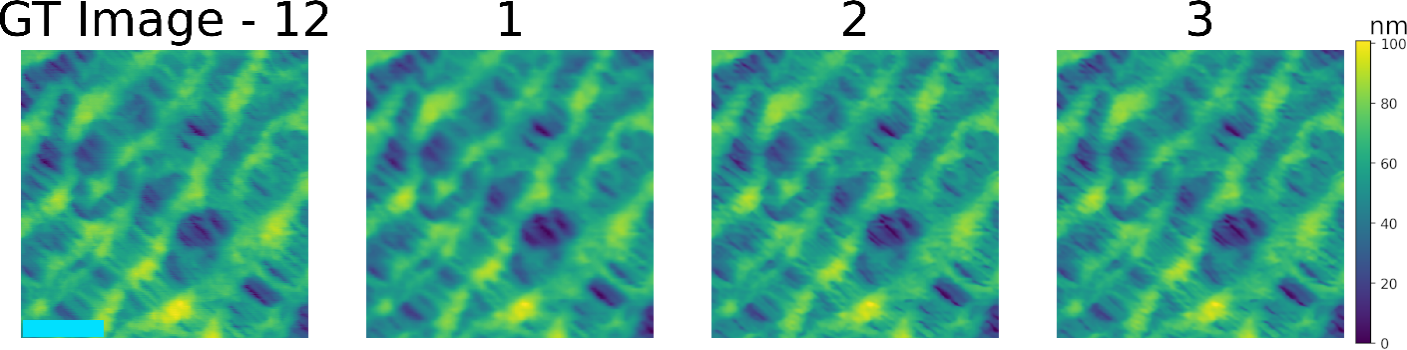
Example of an image set given to AFM experts as part of the survey. The dpi was set to 1000 to ensure that the survey takers could zoom-in to properly assess the results. The left most image is the ground truth image, and the images labeled 1, 2, and 3 are the shuffled outputs of the algorithms used (bicubic interpolation, RCAN, and RDN). The light blue scale bar on left image corresponds to 0.9 μm.

In addition to the algorithmic approaches, three experts in AFM assessed image fidelity and quality. They were provided with all images in each data set. For each image, a set of images was supplied. Each set contained four images aligned in a row. The left most image was the GT image taken experimentally and was labeled as such. The other three (unlabeled) images were all outputs of traditional methods and SR deep learning models: specifically, those from images generated by bicubic interpolation as well as by two deep-learning models (RCAN and RDN). For each image set, the three images were shuffled and labeled 1, 2, and 3. Figure 5 presents an example image that was shown to the AFM experts. The AFM experts did not have the key and, therefore, did not know which of the algorithms corresponded to which of the labeled images for every set. The experts were asked to rank the SR image fidelity and quality from a discrete scale of 1 (lowest) to 6 (highest).

### Statistical analysis

To methodically compare statistical significance between all the various traditional methods and SR deep learning models for each of the metrics listed above, the Tukey’s honestly significant difference (HSD) test [47] performed pairwise comparison of means for the set of all methods and models used. Individual methods and models were compared using box plots, and full comparisons were done using adjacency matrices.

### Code

In this study, all code was written in the Python programming language [48]. In addition to its common packages, we used, OpenCV (v. 4.7.0) [41], PyTorch (v. 2.1.2) [49], PYIQA (v. 0.1.11) [50], Scikit-Image (v. 0.23.2) [51], SciPy (v. 1.13.1) [52], and torchSR github repository for the pre-trained deep learning models [26].

## Data Availability

Data generated and analyzed during this study is currently in use in ongoing research and may be available from the corresponding author upon reasonable request.
